# Optical and photocatalytic properties of TiO_2_ nanoplumes

**DOI:** 10.3762/bjnano.8.20

**Published:** 2017-01-18

**Authors:** Viviana Scuderi, Massimo Zimbone, Maria Miritello, Giuseppe Nicotra, Giuliana Impellizzeri, Vittorio Privitera

**Affiliations:** 1 CNR-IMM, Via S. Sofia 64, 95123 Catania, Italy; 2 CNR-IMM, Z.I. VIII Strada 5, 95121 Catania, Italy

**Keywords:** black titania, nanostructures, photocatalysis, titanium dioxide (TiO_2_)

## Abstract

Here we report the photocatalytic efficiency of hydrogenated TiO_2_ nanoplumes studied by measuring dye degradation in water. Nanoplumes were synthesized by peroxide etching of Ti films with different thicknesses. Structural characterization was carried out by scanning electron microscopy and transmission electron microscopy. We investigated in detail the optical properties of the synthesized material and related them to the efficiency of UV photodegradation of methylene blue dye. The obtained results show that TiO_2_ nanoplumes act as an effective antireflective layer increasing the UV photocatalytic yield of the film.

## Introduction

Today water, energy, and food are the most urgent problems of humanity. Since the seminal work of Honda and Fujishima in 1972 where photo-induced decomposition of water was discovered [1], semiconductor photocatalysis has shown great potential not only for renewable energy generation but also in sustainable technology to remove dangerous contaminants from water [2–6]. In this context, TiO_2_ is one of the most extensively studied materials. However, TiO_2_ is characterized by a wide band gap (ca. 3 eV) resulting in a poor absorption of light in the visible region [7]. Different approaches were proposed to overcome this limit: the inhibition of the recombination of photogenerated electrons and holes [8–9], the increase of the exposed surface area [10–12], and the decrease of the band-gap energy [13–14].

Recently, Chen et al. were able to synthesize black TiO_2_ with a large optical absorption in the visible and infrared region [15–18]. The amorphous shell on a crystalline nanoparticle core, causes the formation of intragap states [19], which are responsible for the high absorption of visible light (and consequently of the black color). Unfortunately, the synthesis of this remarkable material requires high pressures of H_2_ (up to 20 bar) and long annealing treatments (up to 5 days).

Our group investigated the possibility to synthesize black TiO_2_ by an easier method [20]. In 2016 we employed, for the first time [21], hydrogen peroxide etching of Ti films as an easy, rapid, and low-cost method to synthesize hydrogenated TiO_2_ nanoplumes with significant photocatalytic properties under UV and visible light irradiation.

Here we report the optical properties of the TiO_2_ nanoplumes and their correlation with the efficiency of UV photodegradation of methylene blue (MB). The obtained results show that TiO_2_ nanoplumes act as effective antireflective layer increasing the UV photocatalytic yield.

## Results and Discussion

**Figure 1:**
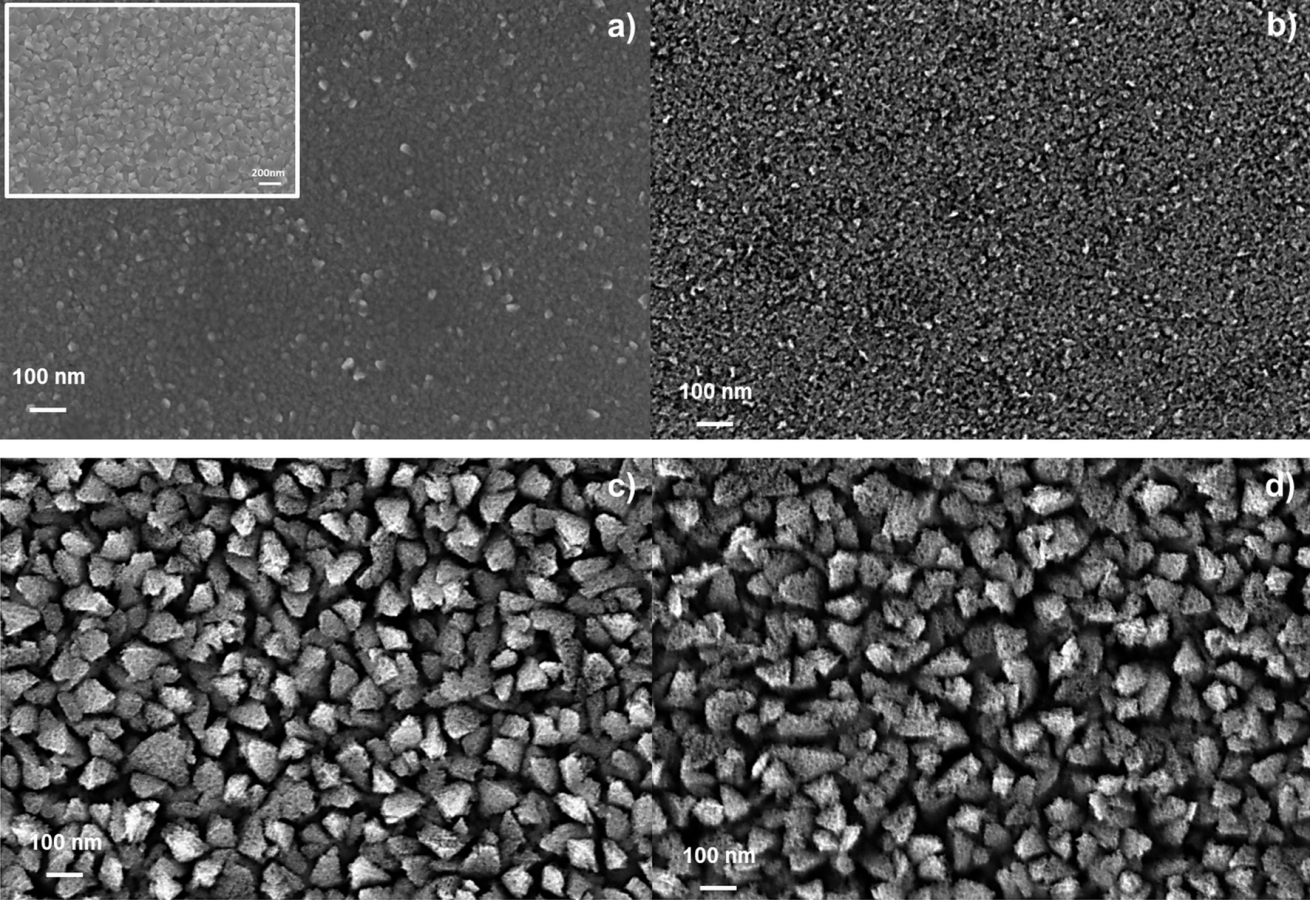
SEM images of a Ti film (70 nm thick) after etching for (a) 60 and (b) 150 s. Images of a Ti film (430 nm thick) after etching for (c) 150 and (d) 190 s. Inset of panel (a): Ti film before the etching.

In order to observe the morphology of the films we analyzed the Ti samples after different etching times by SEM in plan view. Two Ti film thicknesses, 70 nm and 430 nm, were used. Hereafter, we will call the samples Ti (70-*x_i_*) and Ti (430-*x_i_*), where *x_i_* is the time of etching. The images are reported in Figure 1. Figure 1a and Figure 1b show a Ti film of 70 nm after etching for 60 and 150 s, respectively. After 60 s of etching there is no observable change in the surface of the film, compared with the as-deposited Ti film (inset of Figure 1a). After 150 s of etching, instead, the Ti (70-150) sample shows a rough surface. Figure 1c and Figure 1d show a Ti film of 430 nm after etching for 150 and 190 s, respectively. A statistical analysis showed that he roughness of the surface and the porosity of the structure increase with the etching time.

**Figure 2:**
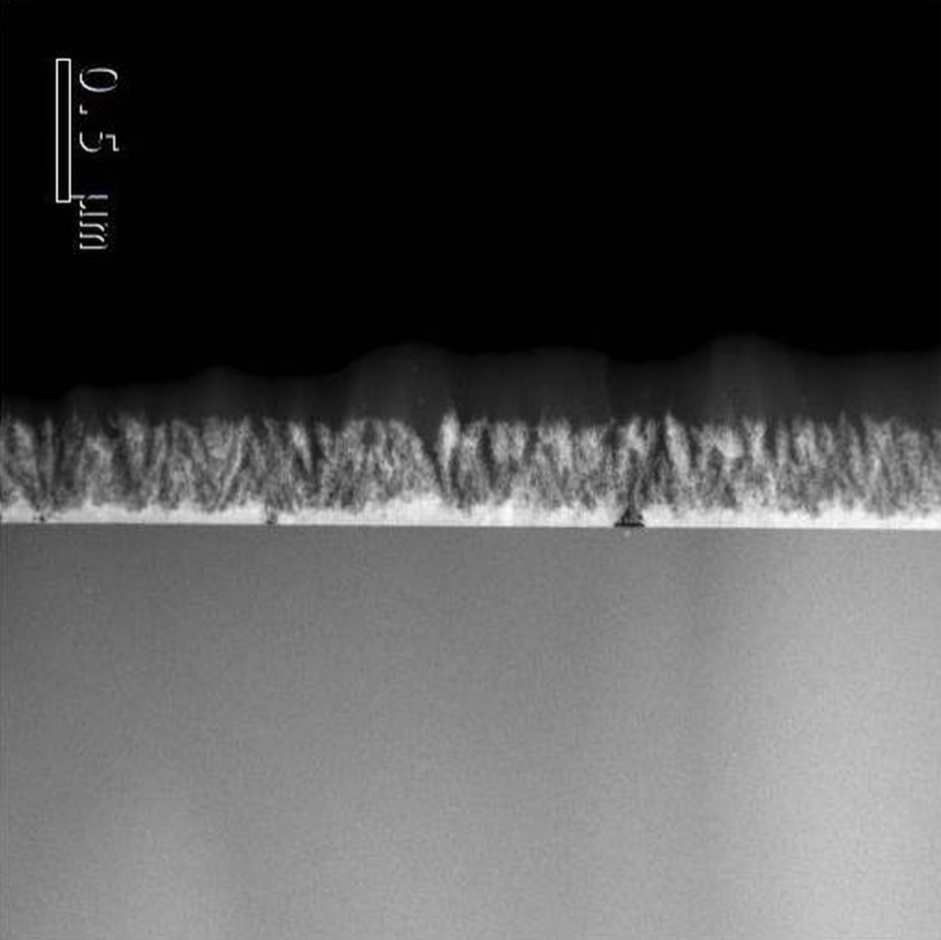
Cross-view TEM image of Ti (430-190).

In Figure 2a, a cross-sectional view STEM image of Ti (430-190) is reported. The sample shows the presence of a nanostructured material. The etching clearly expands from the surface of the Ti film to the bulk of the material, generating a nanostructured film with high porosity and roughness, that we called nanoplumes [21]. Nanoplumes (ca. 300 nm in thickness) show the presence of a residual Ti layer at the bottom (ca. 70 nm in thickness). Energy dispersive X-ray analyses reported in [21] indicated that the nanoplumes are made of TiO_2_. In addition, an accurate characterization of the nanoplumes performed by X-ray diffraction (XRD) measurements, X-ray Fourier transform infrared spectroscopy (FTIR), and photoelectron spectroscopy (XPS), revealed that after the etching the samples are amorphous and composed of titanium and oxygen [21]. An abundance of –OH groups was revealed on the surface of the samples.

**Figure 3:**
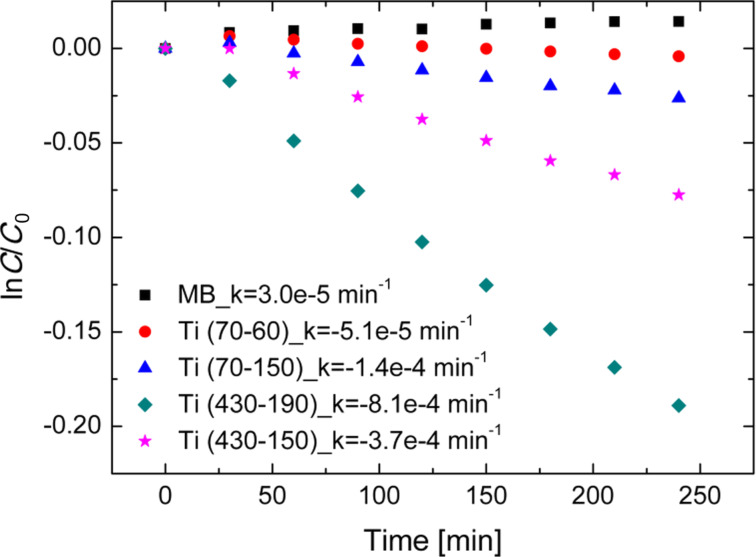
MB degradation under UV light irradiation for five samples: MB (black squares), MB with Ti (70-60) (red circles), MB with Ti (70-150) (blue triangles), MB with Ti (430-150) (pink stars), MB with Ti (430-190) (green diamonds).

Figure 3 shows the logarithmic residual concentration of MB as a function of the time under UV irradiation. *C* is the measured concentration of MB during irradiation, and *C*_0_ is the starting concentration of MB. A pseudo-first order photocatalytic rate constant, *k* [3], was extracted and the values are reported in Figure 3 for all the analyzed samples. The surfaces were previously saturated with MB molecules. The samples were kept immersed in the MB solution in dark for 12 h. So the surfaces reached a steady-state of adsorption [21]. No photobleaching of the MB molecules on the control solution was detected, therefore the decay in the MB concentration is only related to the degradation of the reactant through a photocatalytic reaction with the surface. Ti (430-190) samples exhibit the best photocatalytic activity in degrading the MB dye under UV irradiation. In order to analyze the obtained photocatalytic results, it is necessary to take into account different factors that influence the reaction. Although the macroscopic surface is the same, the active surface areas for the photocatalytic reaction is different for each type of sample. Moreover the dynamic mechanisms of liquid circulation around [22] and inside nanostructures are not obvious [23]. However, the possible enhancement in the light trapping efficiency could provide additional photogenerated carriers, and as a consequence could favor the photocatalytic activity.

**Figure 4:**
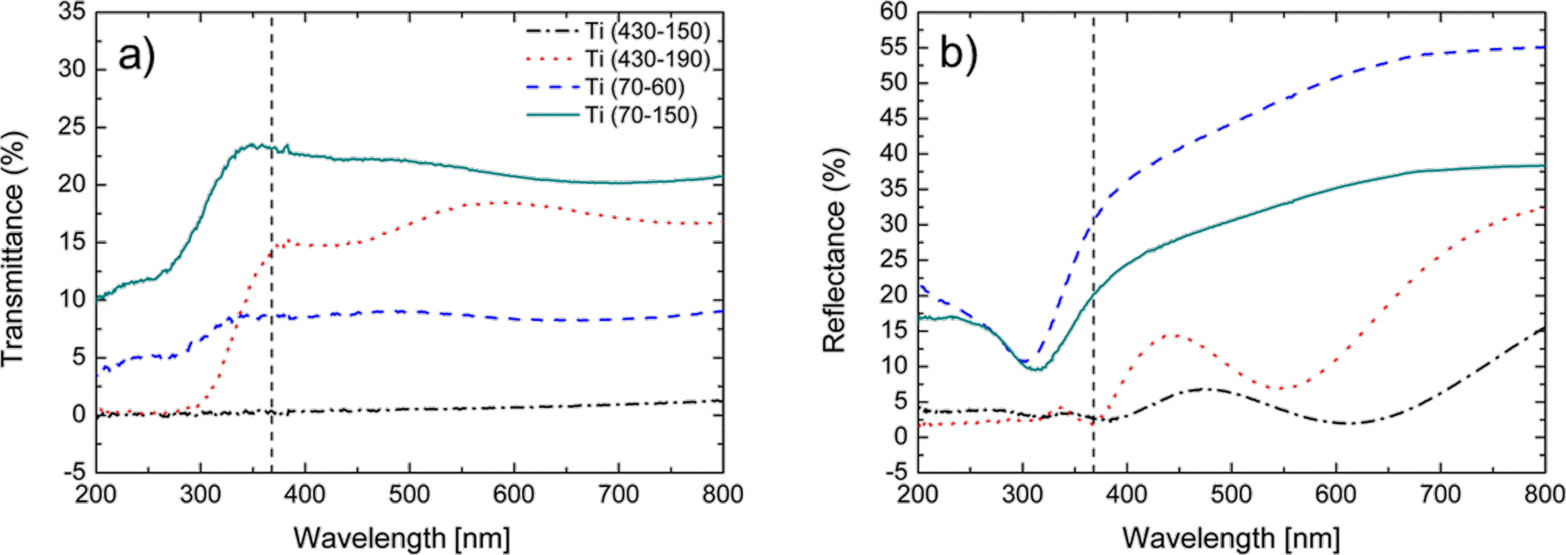
Transmittance (a) and reflectance (b) measurements in the range of 200–800 nm. The vertical lines mark the wavelength of the UV-A light source used for the photocatalytic experiments.

In order to gain more insight into the realized structure, we performed transmittance (*T*) and reflectance (*R*) measurements in the range of 200–800 nm. The optical spectra are reported in Figure 4. The transmittance spectra (Figure 4a) show that the transmitted light by the samples is closely related to the thickness of the residual Ti layer and, consequently, to the etching time. In fact, for a fixed Ti starting thickness we observed a more efficient transmittance after longer etching times (compare the dashed-dotted with the dotted line, and the dashed with the continuous line).

The reflectance measurements are reported in Figure 4b. The total reflectance spectra of Ti (70-60) and Ti (70-150) present a shift in the reflectance peak, from 302 to 314 nm. This shift may be related to the variation on the sample thickness, due to the different chemical etching time. Although the measured spectra include wavelengths from 200 to 800 nm we will focus on the UV region, since the light source of photocatalytic experiments emits in this range (dashed vertical line). In this region the absolute values of reflectance for Ti (70-60) and Ti (70-150) are about 10%. The total reflectance of Ti (430-150) and Ti (430-190) exhibits an almost completely suppression, below 5% in the UV range, and a 50% decrease between 410 and 700 nm compared to the previous two samples. The oscillations of the spectra are due to optical interference. Ti (430-190) exhibits a high exposed surface area due to the longer chemical etching and the lowest reflectivity in the UV-A region. Both parameters affect the photocatalytic properties of the sample making it particularly active in the presence of MB.

**Figure 5:**
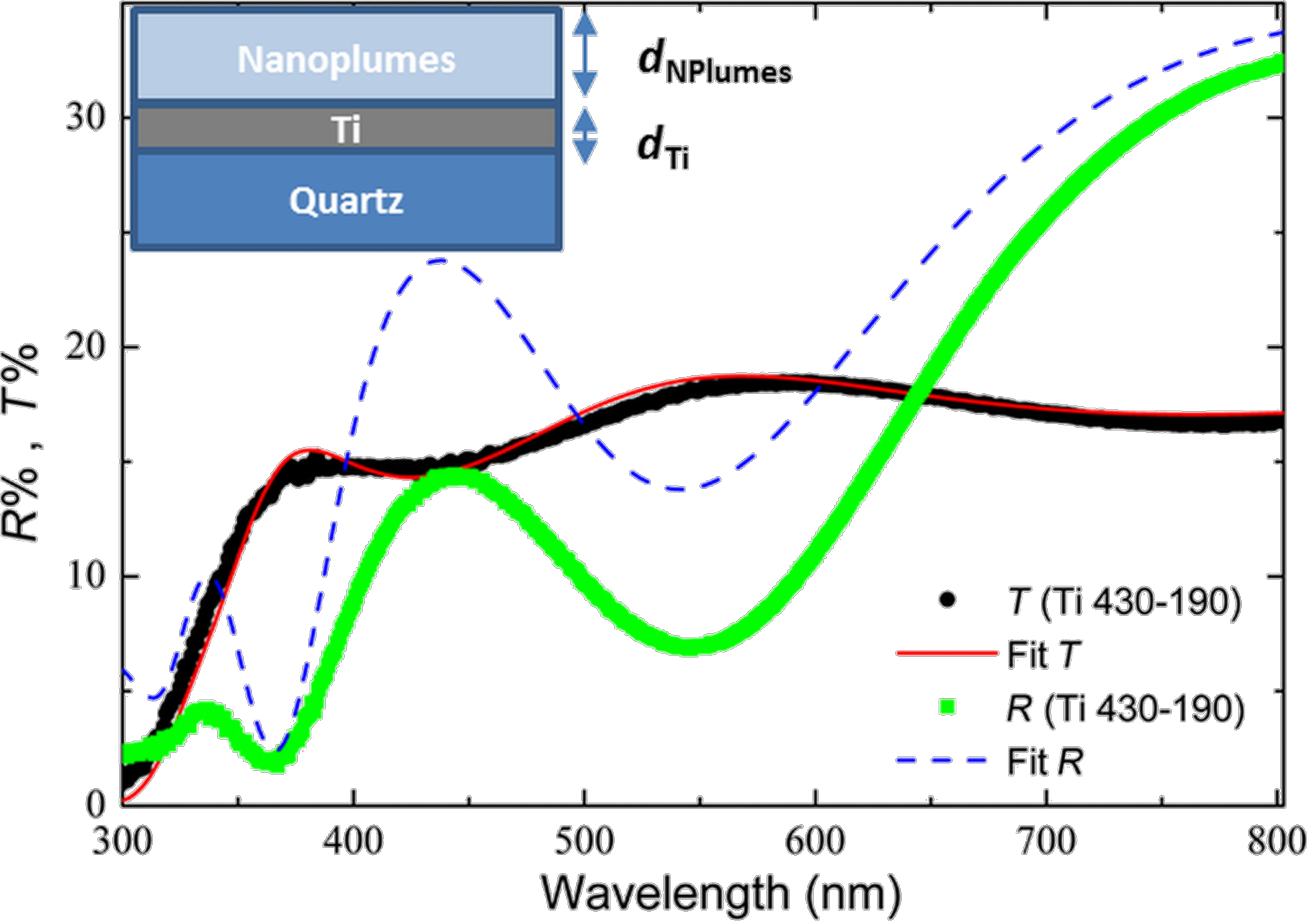
Fitting of the transmittance and reflectance spectra of Ti (430-190).

As shown before [21], the defects introduced in the material by the chemical etching (in particular, Ti^3+^ and OH groups) are responsible for a blurring of the valence and conduction bands. Consequently, a reduction of the optical gap was reported. Ascertained of the high photocatalytic activity of the Ti (430-190) sample due to its structural and optical properties, we interpreted the *R* and *T* measurements in terms of the Fresnel formulae [24], regardless of the effects of depolarization due to the roughness and non-uniformity of the surface. We assumed that the sample is constituted by two layers on a quartz substrate, namely a titanium oxide layer and a metallic titanium layer (from the top to the bottom). A schematic view is shown as inset in Figure 5. The refractive indexes of the three layers (TiO_2_/Ti/quartz) were calculated separately. For the quartz substrate it was assumed to be 1.5 [25]. The refractive index of the metallic film was extracted by fitting the reflectance spectrum of the titanium layer before the chemical etching. We assumed that the functional form for the dielectric constant of the metallic film is given by the “Drude free carrier” expression [26]:

[1]
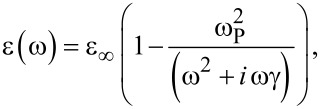


where ω, ω_P_, γ and ε_∞_ are, respectively, the light frequency, the plasma frequency, the damping constant, and the low-frequency dielectric constant tabulated for titanium. The refractive index of titanium is calculated by the square root of Equation 1 [26]:

[2]
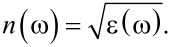


The refractive index of the TiO_2_ film was extracted by fitting both the reflectance and transmittance spectra of the Ti (430-190) sample, by using a Forouhi–Bloomer (FB) functional form for amorphous samples [27–29]:

[3]
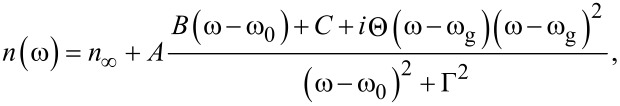


where

[4]
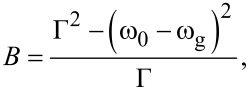


[5]
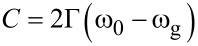


and Θ(ω − ω_g_) is a step function, ω_g_ is the energy gap of the amorphous material, *n*_∞_, *A*, ω_0_, Γ are the “low-frequency” refractive index, the amplitude, the position, and the damping constant of the FB oscillator, respectively. The transmittance spectra reported in Figure 4a clearly indicate that the transmittance strongly depends on the thickness of the metallic film (*d*_m_), whereas the reflectance spectra are mostly influenced by the TiO_2_ layer parameters *n*_∞_, ω_g_, *A*, ω_0_, Γ, and *d*_0_ (thickness of titanium oxide layer). To start the fitting, we fixed *d*_0_ and *d*_m_ to be 300 and 70 nm, respectively, as obtained by TEM analysis, and we assumed values of ω_g_ and ω_0_ of 3.0 eV and 4.3 eV, respectively, in agreement with the values reported for TiO_2_ [30]. The other parameters *n*_∞_, *A*, and Γ were extracted by fitting both transmittance and reflectance. The simulated reflectance and transmittance spectra by the Fresnel formulae [24] are shown in Figure 5 by the dashed and the continuous line, respectively.

We wish to underline that the position of the maxima and minima in the measured reflectance and transmittance spectra do not coincide as expected. For example, the Ti (430-190) sample shows a minimum of reflectance at 547 nm, which is shifted by about 40 nm with respect to the maximum of the transmittance (at 592 nm). This effect is related to the intrinsic inhomogeneity of the etching process. Although the shape of both *T* and *R* spectra can be reproduced by the fitting process, some minor discrepancies between the experimental and calculated spectra need to be addressed. The fitted transmittance is in perfect agreement with the experimental spectrum (Figure 5). Indeed, the fit mainly depends on the thickness [27–29] of the metallic Ti film and this value was established by the TEM analyses. On the other hand, the fit of the reflectance is less accurate at shorter wavelengths and in particular in the UV range. This discrepancy can be explained by considering the scattering effects from a rough surface. The light reflected by the titanium layer undergoes multiple scattering in the nanoplume layer and it is partially re-absorbed. This effect is due to the high roughness and porosity of the material. Therefore, the measured spectrum shows a lower reflectivity value compared to the fitted spectrum in which the scattering effects were not considered.

It is worth noting that the refractive index of the nanoplumes, albeit it depends on the wavelength, does not exceed the value of 1.4. In particular, it is 1.2 at 800 nm, and 1.3 at 300 nm. TiO_2_ is known to be a high refractive index material (*n* = 2.5–2.7) [31], whereas the nanoplumes show a very low refractive index. This result can be correlated to the high porosity and the high content of air into the nanoplumes, so that the refractive index can be considered as an average value of those of air and TiO_2_.

The model used to fit the experimental *R* and *T* curves shows that the metal Ti layer acts as an efficient reflective layer, whereas the nanoplume layer acts as an antireflective coating. We found an ideal combination of 70 nm of highly reflective metallic Ti under 300 nm of TiO_2_ with low refractive index. Moreover, scattering effects improve the light adsorption by the nanoplumes, increasing the generation of electron–hole pairs and, therefore, enhancing the photocatalytic performance.

## Conclusion

In summary, TiO_2_ nanoplumes were synthesized by a straightforward method, involving rapid chemical etching of Ti films in a H_2_O_2_ solution. The present results reveal that the most important optical effect of the synthesized nanoplumes is the suppression of reflectance, particularly in the UV range of the spectra. The suppression of the reflectance is due to scattering effects of the nanoplumes, which lead to the absorption of additional photons reflected from titanium layer. The generation of electron–hole pairs is subsequently increased, and leads to the significant photocatalytic activity of the TiO_2_ nanoplumes.

## Experimental

The samples were synthesized by chemical etching of titanium films as reported in [21]. Briefly, titanium films with a thickness of about 70 and 430 nm, were sputtered on quartz substrates by ultra-high vacuum magnetron sputtering from a Ti target of 99.99% purity. The Ti samples (1 cm × 1 cm in size) were put into 3 mL H_2_O_2_ (30%) solution at 60 °C for different etching times. Afterwards, the samples were rinsed with deionized water and dried in air.

SEM analyses in plan view were performed by a field emission Zeiss Supra 25 microscope. TEM analyses were performed in cross-sectional view with a JEOL JEM ARM200CF in scanning mode (STEM) at 60 kV of beam acceleration voltage using the microscope installed at the Beyond-Nano laboratory in Catania (Italy). The optical characterization was obtained by extracting both the total transmittance (*T*) and the total reflectance (*R*) spectra in the 200–800 nm wavelength range, by using a spectrophotometer Perkin-Elmer Lambda 35 equipped with an integrating sphere (RSA-PE-20, Perking-Elmer).

The photocatalytic properties of the synthesized material were evaluated by the degradation of methylene blue (MB) dye, a chemical compound commonly used to evaluate the photocatalytic efficiency of a material. The experimental setup was in agreement with the ISO 10678:2010 international standard. The samples were immersed in a solution (2 mL) containing MB and de-ionized water (starting concentration: 1.3 × 10^−5^ M). The mixture was irradiated by an 8 W UV lamp (350–400 nm wavelength range) with a irradiance of 1 mW/cm^2^, for a total time of 3 h. Every 30 min of irradiation the solutions were measured with an UV–vis spectrophotometer (Perkin-Elmer Lambda 45) in the 500–800 nm wavelength range. The degradation of MB was evaluated by the absorbance peak at 664 nm, according to the Lambert–Beer law. The decomposition of the dye in absence of any material was also measured as a reference. Before each measurement, the samples were irradiated by the UV lamp for 50 min in order to remove the hydrocarbons localized on the sample surface [32].
